# Supplementary material for: Pregnancy loss and risk of multiple sclerosis and autoimmune neurological disorder: A nationwide cohort study
Source: PLoS One. 2022 Mar 31;17(3):e0266203. doi: 10.1371/journal.pone.0266203 (PMC8970484; doi:10.1371/journal.pone.0266203)

**S1 Fig. Prior and posterior probability density.**

Legend: Bayesian inference estimates the posterior distribution using data, prior knowledge, and a statistical model. The current study aimed to assess if exposure to pregnancy loss (categories: 0, 1, 2, ≥3 non-consecutive losses, primary recurrent pregnancy loss, and secondary pregnancy loss) was associated with developing multiple sclerosis. In the primary model a modestly informative prior was chosen, using a Gaussian distribution with mean Incidence Rate Ratio (IRR) of 1, and 95% of the probability mass within IRRs between 0.25 and 4 (red distributions). Thus, no association was a priori assumed to be most likely, however, small or large IRR were less likely, but not impossible. The main model was a Poisson regression which combined information from the priors and data from the ~1.5 million women in the cohort and explored the posterior distribution. The exponentiated posterior distribution corresponded to the adjusted IRR of multiple sclerosis after exposure to pregnancy loss (light blue posterior distributions) as compared to no pregnancy losses. The IRR was then summarized as the median and 95% highest posterior density interval, termed credible interval (CI), shown in light blue text.


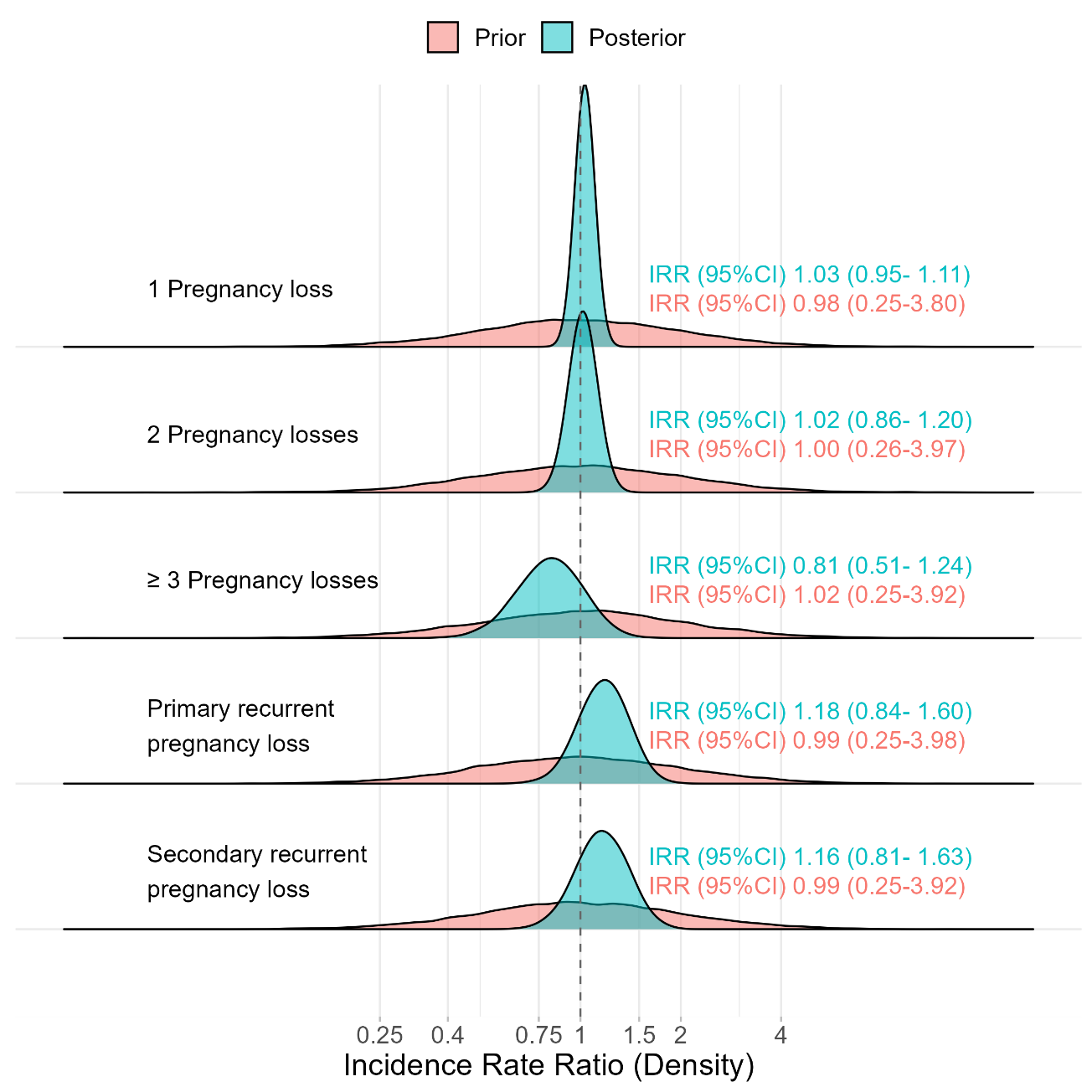

Supplement: S1 Fig — (DOCX) [file pone.0266203.s001.docx]
