# Supplementary material for: Pregnancy loss and risk of multiple sclerosis and autoimmune neurological disorder: A nationwide cohort study
Source: PLoS One. 2022 Mar 31;17(3):e0266203. doi: 10.1371/journal.pone.0266203 (PMC8970484; doi:10.1371/journal.pone.0266203)
Supplement: S1 Table — (DOCX) [file pone.0266203.s002.docx]

| S1 Table. Registers and definitions used. | | | | |
| --- | --- | --- | --- | --- |
| **Variable** | **Register** | **Codes** | **Note** | **Missing ^a^, %** |
| Outcome | | | | |
| Multiple sclerosis | MSR |  | Variables diagnosis date and onset date | 0 |
| Amyotrophic lateral sclerosis | NPR | ICD-8: 3480  ICD-10: G122 | Incident primary or secondary diagnosis | 0 |
| Gillian-Barré syndrome | NPR | ICD-8: 35400  ICD-10: G610 | Incident primary or secondary diagnosis | 0 |
| Myasthenia gravis | NPR | ICD-8: 73309  ICD-10: G700 | Incident primary or secondary diagnosis | 0 |
| Exposure | | | | |
| Pregnancy loss | NPR, MBR | ICD-8: 6346, 6438, 6439, 6451  ICD-10: O020, O021, O03 |  | 0 |
| Recurrent pregnancy loss | NPR, MBR | ICD-8: 6430, Y6439  ICD-10: N96, O262 | Either specific diagnosis or 3 consecutive pregnancy losses (as defined above) with no live births or induced abortions in between | 0 |
| Stillbirth | MBR |  | Individual variable | 0 |
| Other | | | | |
| Date of birth, death, immigration, emigration, and kinship | CRS |  | Individual variable | 0 |
| Obtained bachelor’s degree or higher educational degree | DST |  | Individual variable | 3.7 |
| Family history of multiple sclerosis | NPR | ICD-8: 340  ICD-10: G35 | Incident primary or secondary diagnosis | 9.3 |
| Smoking status | MBR |  | Individual variable | 47.6 |
| Alternative smoking definition (S2 Table) | NPR, DPR | ICD-10: F17  ATC: N07BA | Incident code | 0 |
| *Abbreviations:* ATC: Anatomical Therapeutic Chemical Classification system; CRS: Danish Civil Registration System; DST: Demographic Registers of Statistics Denmark; ICD-8: International Classification of Disease and Health Related Problems, 8^th^ revision; ICD-10: 10^th^ revision; MBR: Danish Medical Birth Register; MSR: Danish Multiple Sclerosis Register; NPR: Danish National Patient Register; DPR: Danish Prescription Register  ^a^ Percent of total cohort with missing or unknown data | | | | |
