# Supplementary material for: Pregnancy loss and risk of multiple sclerosis and autoimmune neurological disorder: A nationwide cohort study
Source: PLoS One. 2022 Mar 31;17(3):e0266203. doi: 10.1371/journal.pone.0266203 (PMC8970484; doi:10.1371/journal.pone.0266203)
Supplement: S2 Table — (DOCX) [file pone.0266203.s003.docx]

| S2 Table. Data set used to create Fig 1 | | | | | |  |
| --- | --- | --- | --- | --- | --- | --- |
| **Number in study**  **by age during follow-up** | **0 PL** | **1 PL** | **2 PL** | **≥3 non-consecutive PL** | **Primary RPL** | **Secondary RPL** |
| 12 | 1125801 | <5 | <5 | <5 | <5 | <5 |
| 13 | 1170646 | <5 | <5 | <5 | <5 | <5 |
| 14 | 1215483 | 28 | <5 | <5 | <5 | <5 |
| 15 | 1258242 | 128 | <5 | <5 | <5 | <5 |
| 16 | 1301110 | 566 | 4 | <5 | <5 | <5 |
| 17 | 1344274 | 1460 | 20 | <5 | 9 | <5 |
| 18 | 1383308 | 3225 | 80 | <5 | 23 | <5 |
| 19 | 1422438 | 6528 | 235 | <5 | 66 | 5 |
| 20 | 1466758 | 11255 | 534 | 10 | 147 | 14 |
| 21 | 1412281 | 16992 | 1045 | 20 | 279 | 43 |
| 22 | 1360890 | 23362 | 1691 | 50 | 451 | 87 |
| 23 | 1309432 | 30280 | 2520 | 89 | 641 | 163 |
| 24 | 1257673 | 38117 | 3502 | 151 | 882 | 266 |
| 25 | 1206565 | 46907 | 4619 | 247 | 1150 | 422 |
| 26 | 1154633 | 56685 | 6036 | 361 | 1455 | 578 |
| 27 | 1103930 | 66886 | 7508 | 490 | 1808 | 812 |
| 28 | 1053533 | 77197 | 9154 | 659 | 2142 | 1062 |
| 29 | 1005143 | 87106 | 10972 | 849 | 2407 | 1346 |
| 30 | 959018 | 96332 | 12780 | 1043 | 2674 | 1669 |
| 31 | 916063 | 104521 | 14405 | 1293 | 2931 | 2006 |
| 32 | 875312 | 111174 | 16102 | 1550 | 3165 | 2332 |
| 33 | 837159 | 116417 | 17604 | 1805 | 3350 | 2641 |
| 34 | 801779 | 120529 | 18936 | 1998 | 3501 | 2953 |
| 35 | 768648 | 123120 | 20186 | 2215 | 3612 | 3206 |
| 36 | 736410 | 124651 | 21149 | 2433 | 3693 | 3452 |
| 37 | 705616 | 124880 | 21857 | 2626 | 3747 | 3694 |
| 38 | 674764 | 124097 | 22377 | 2778 | 3760 | 3859 |
| 39 | 644541 | 122398 | 22589 | 2889 | 3744 | 4000 |
| 40 | 614505 | 119700 | 22691 | 2995 | 3692 | 4035 |
| 41 | 585731 | 116331 | 22487 | 3063 | 3620 | 4063 |
| 42 | 557093 | 112243 | 22047 | 3034 | 3505 | 4023 |
| 43 | 526793 | 107016 | 21330 | 2986 | 3337 | 3896 |
| 44 | 497602 | 101597 | 20459 | 2918 | 3187 | 3708 |
| 45 | 469141 | 95962 | 19424 | 2806 | 2993 | 3503 |
| 46 | 439450 | 90011 | 18270 | 2637 | 2807 | 3279 |
| 47 | 410623 | 84130 | 17056 | 2456 | 2603 | 3018 |
| 48 | 382952 | 78166 | 15853 | 2277 | 2423 | 2793 |
| 49 | 355559 | 72292 | 14690 | 2107 | 2218 | 2572 |
| 50 | 327322 | 66111 | 13426 | 1893 | 2026 | 2350 |
| 51 | 296646 | 59384 | 12036 | 1709 | 1835 | 2120 |
| 52 | 263424 | 52273 | 10558 | 1490 | 1616 | 1866 |
| 53 | 231171 | 45588 | 9209 | 1277 | 1409 | 1619 |
| 54 | 200100 | 39033 | 7845 | 1076 | 1203 | 1374 |
| 55 | 169230 | 32573 | 6549 | 896 | 999 | 1162 |
| 56 | 139835 | 26558 | 5299 | 721 | 830 | 962 |
| 57 | 111197 | 20865 | 4121 | 535 | 658 | 756 |
| 58 | 82856 | 15224 | 2985 | 385 | 487 | 558 |
| 59 | 55308 | 9993 | 1892 | 249 | 338 | 345 |
| 60 | 27500 | 4845 | 908 | 114 | 185 | 169 |
| *Abbreviations*: PL: Pregnancy loss; RPL: Recurrent pregnancy loss; <5: Number below 5, masked due to legal restrictions | | | | | | |
