# Supplementary material for: Pregnancy loss and risk of multiple sclerosis and autoimmune neurological disorder: A nationwide cohort study
Source: PLoS One. 2022 Mar 31;17(3):e0266203. doi: 10.1371/journal.pone.0266203 (PMC8970484; doi:10.1371/journal.pone.0266203)
Supplement: S3 Table — (DOCX) [file pone.0266203.s004.docx]

| S3 Table. Association of Pregnancy loss with Multiple Sclerosis, further adjusted for smoking status defined by hospital admission for smoking (ICD-10: F17) or fulfilling prescription for a smoking cessation drug (ATC: N07AB). | | | | | |
| --- | --- | --- | --- | --- | --- |
| **Analysis**  **Pregnancy loss exposure ^a^** | **Events** | **Person-years** | **Incidence rate ^b^** | **Crude IRR ^c^ (95% CI)** | **Adjusted IRR ^c, d^ (95% CI)** |
|  | n = 1,513,544 (100%) | | | | |
| 0 | 6,654 | 36,656,369 | 1.82 | 1 | 1 |
| 1 | 807 | 2,986,620 | 2.70 | 1.49 (1.38-1.60) | 1.03 (0.95-1.11) |
| 2 | 137 | 505,490 | 2.71 | 1.48 (1.25-1.75) | 1.02 (0.86-1.20) |
| ≥3 non-consecutive ^e^ | 12 | 61,249 | 1.96 | 1.06 (0.60-1.73) | 0.75 (0.42-1.24) |
| Primary RPL ^f^ | 30 | 87,626 | 3.42 | 1.80 (1.24-2.52) | 1.21 (0.83-1.71) |
| Secondary RPL ^f^ | 27 | 82,839 | 3.26 | 1.71 (1.15-2.44) | 1.20 (0.81-1.71) |
| *Abbreviations:* ATC: Anatomical Therapeutic Chemical Classification System, ICD: International Statistical Classification of Diseases and Related Health Problems; CI, credible interval; IRR, incidence rate ratio;  RPL: recurrent pregnancy loss  ^a^ Pregnancy loss (i.e. miscarriage) was defined hierarchically with no pregnancy losses at the lowest level and recurrent pregnancy loss at the highest level.  ^b^ Incidence rate per 10,000 person-years.  ^c^ Estimated using a Poisson model  ^d^ Estimates adjusted for the number of live births, obtained bachelor’s degree, family history of multiple sclerosis, calendar period, and age group unless otherwise stated.  ^e^ ≥3 non-consecutive pregnancy losses (not fulfilling criteria for recurrent pregnancy loss).  ^f^ Recurrent pregnancy loss (RPL) defined as three consecutive pregnancy losses, either preceded by a delivery (secondary) or not (primary). | | | | | |
