# Supplementary material for: Pregnancy loss and risk of multiple sclerosis and autoimmune neurological disorder: A nationwide cohort study
Source: PLoS One. 2022 Mar 31;17(3):e0266203. doi: 10.1371/journal.pone.0266203 (PMC8970484; doi:10.1371/journal.pone.0266203)
Supplement: S1 Appendix — (DOCX) [file pone.0266203.s005.docx]

**S1 Appendix. Restriction periods between pregnancies**

As pregnancies could lead to multiple hospital contacts during clinical contact, a set of restriction periods were used to ascertain each pregnancy was only counted once. In case two pregnancies were overlapping, the first was kept.

- At least 90 days between two complications of early pregnancy (i.e. pregnancy loss, induced abortion, extrauterine pregnancy, or molar pregnancy)
- At least 154 days between a complication of early pregnancy and a succeeding live or stillbirth.
- At least 60 days between a live or stillbirth and a succeeding complication of early pregnancy.
- At least 154 days between a live or stillbirth and a succeeding live or stillbirth.
