# Supplementary material for: Pregnancy loss and risk of multiple sclerosis and autoimmune neurological disorder: A nationwide cohort study
Source: PLoS One. 2022 Mar 31;17(3):e0266203. doi: 10.1371/journal.pone.0266203 (PMC8970484; doi:10.1371/journal.pone.0266203)
Supplement: S2 Appendix — (DOCX) [file pone.0266203.s006.docx]

**S2 Appendix. Specification of the Bayesian model**

Bayesian models were fit using Stan software which uses a Hamiltonian Monte Carlo algorithm to explore the posterior distribution. The main statistical model chosen was a Poisson regression while a secondary analysis used a negative binomial regression. In the primary analysis, a modestly informative prior was chosen, and the intercept was set to a Gaussian distribution with mean 0 and standard deviation 2.5, and for all parameters the prior was set to a Gaussian distribution with mean 0 and standard deviation 0.7. This corresponded to 95% of the probability mass of the IRR was between 0.25 to 4, and an IRR of 1 was the most likely a priori. Four Markov chains were run for 10,000 iterations, (including 5,000 warm-up iterations which were discarded) in each analysis. Multivariable analyses were run applying a scaled QR decomposition to the design matrix. Convergence of the four chains was confirmed in trace plots and by calculating the R-hat statistic.
