# Supplementary material for: Pregnancy loss and risk of multiple sclerosis and autoimmune neurological disorder: A nationwide cohort study
Source: PLoS One. 2022 Mar 31;17(3):e0266203. doi: 10.1371/journal.pone.0266203 (PMC8970484; doi:10.1371/journal.pone.0266203)
Supplement: Attachment — Submitted filename: Response to Reviewers.docx [file pone.0266203.s007.docx]

**JOURNAL REQUIREMENTS**

**1. When submitting your revision, we need you to address these additional requirements.**

**Please ensure that your manuscript meets PLOS ONE's style requirements, including those for file naming. The PLOS ONE style templates can be found at** 
[**https://journals.plos.org/plosone/s/file?id=wjVg/PLOSOne_formatting_sample_main_body.pdf**](https://journals.plos.org/plosone/s/file?id=wjVg/PLOSOne_formatting_sample_main_body.pdf) **and** 
[**https://journals.plos.org/plosone/s/file?id=ba62/PLOSOne_formatting_sample_title_authors_affiliations.pdf**](https://journals.plos.org/plosone/s/file?id=ba62/PLOSOne_formatting_sample_title_authors_affiliations.pdf)

We have carefully ensured the manuscript meets PLOS ONE’s style requirements.

**2. Thank you for stating in your Funding Statement: 
(This work was supported by The Research Fund of Rigshospitalet, Copenhagen University Hospital [grant number E-22515-01] awarded to APM and ØL. URL:** [**https://www.forskningspuljer-rh.dk/**](https://www.forskningspuljer-rh.dk/) **Ole Kirks Foundation [no grant number] awarded to HSN. URL:** [**https://www.olekirksfond.dk/**](https://www.olekirksfond.dk/)**)
Please provide an amended statement that declares *all* the funding or sources of support (whether external or internal to your organization) received during this study, as detailed online in our guide for authors at** [**http://journals.plos.org/plosone/s/submit-now**](http://journals.plos.org/plosone/s/submit-now)**.  Please also include the statement “There was no additional external funding received for this study.” in your updated Funding Statement. 
Please include your amended Funding Statement within your cover letter. We will change the online submission form on your behalf.**

We have included the amended funding statement in the cover letter.

**3. Thank you for stating the following in the Competing Interests section: 
(I have read the journal's policy and the authors of this manuscript have the following competing interests: The authors report no conflicts of interest for the submitted research. HSN reports grants from Freya Biosciences ApS, Ferring Pharmaceuticals, BioInnovation Institute, Ministry of Education, Novo Nordisk Foundation, Augustinus Foundation, Oda and Hans Svenningsens Foundation and honoraria from Ferring Pharmaceuticals, Merck A/S, Astra Zenica, Cook Medical, and DW reports a grant from Novo Nordisk Foundation during the conduct of this study.)**

**We note that you received funding from a commercial source: (Freya Biosciences ApS, Ferring Pharmaceuticals, BioInnovation Institute, Ministry of Education, Novo Nordisk Foundation, Augustinus Foundation, Oda and Hans Svenningsens Foundation, Merck A/S, Astra Zenica, Cook Medical,Novo Nordisk)**

**Please provide an amended Competing Interests Statement that explicitly states this commercial funder, along with any other relevant declarations relating to employment, consultancy, patents, products in development, marketed products, etc.**

**Within this Competing Interests Statement, please confirm that this does not alter your adherence to all PLOS ONE policies on sharing data and materials by including the following statement: "This does not alter our adherence to PLOS ONE policies on sharing data and materials.” (as detailed online in our guide for authors** [**http://journals.plos.org/plosone/s/competing-interests**](http://journals.plos.org/plosone/s/competing-interests)**).  If there are restrictions on sharing of data and/or materials, please state these. Please note that we cannot proceed with consideration of your article until this information has been declared.**

**Please include your amended Competing Interests Statement within your cover letter. We will change the online submission form on your behalf.**

We have included the amended funding statement in the cover letter.

**4. In your Data Availability statement, you have not specified where the minimal data set underlying the results described in your manuscript can be found. PLOS defines a study's minimal data set as the underlying data used to reach the conclusions drawn in the manuscript and any additional data required to replicate the reported study findings in their entirety. All PLOS journals require that the minimal data set be made fully available. For more information about our data policy, please see** [**http://journals.plos.org/plosone/s/data-availability**](http://journals.plos.org/plosone/s/data-availability)**.**

**Upon re-submitting your revised manuscript, please upload your study’s minimal underlying data set as either Supporting Information files or to a stable, public repository and include the relevant URLs, DOIs, or accession numbers within your revised cover letter. For a list of acceptable repositories, please see** [**http://journals.plos.org/plosone/s/data-availability#loc-recommended-repositories**](http://journals.plos.org/plosone/s/data-availability#loc-recommended-repositories)**. Any potentially identifying patient information must be fully anonymized.**

**Important: If there are ethical or legal restrictions to sharing your data publicly, please explain these restrictions in detail. Please see our guidelines for more information on what we consider unacceptable restrictions to publicly sharing data:** [**http://journals.plos.org/plosone/s/data-availability#loc-unacceptable-data-access-restrictions**](http://journals.plos.org/plosone/s/data-availability#loc-unacceptable-data-access-restrictions)**. Note that it is not acceptable for the authors to be the sole named individuals responsible for ensuring data access.**

**We will update your Data Availability statement to reflect the information you provide in your cover letter.**

The data used in the current study cannot be made public due to Danish legal restrictions which prohibits export of individual level data from nationwide registers. The minimal sufficient dataset is all included in the manuscript, except for the data used to create Figure 1. This data has now been included in the Supporting information as S3 Table. We have included an amended Data Availability statement in the cover letter.

**5. Please review your reference list to ensure that it is complete and correct. If you have cited papers that have been retracted, please include the rationale for doing so in the manuscript text, or remove these references and replace them with relevant current references. Any changes to the reference list should be mentioned in the rebuttal letter that accompanies your revised manuscript. If you need to cite a retracted article, indicate the article’s retracted status in the References list and also include a citation and full reference for the retraction notice.**

We have carefully reviewed the reference list and ensured it is complete and correct. We have not cited any retracted papers and no changes have been made.

**REVIEWERS COMMENTS**

Reviewer's Responses to Questions

**Comments to the Author**

**1. Is the manuscript technically sound, and do the data support the conclusions?

The manuscript must describe a technically sound piece of scientific research with data that supports the conclusions. Experiments must have been conducted rigorously, with appropriate controls, replication, and sample sizes. The conclusions must be drawn appropriately based on the data presented.**

**Reviewer #1: Yes**

**Reviewer #2: Yes**

**2. Has the statistical analysis been performed appropriately and rigorously?**

**Reviewer #1: Yes**

**Reviewer #2: Yes**

**3. Have the authors made all data underlying the findings in their manuscript fully available?

The** [**PLOS Data policy**](http://www.plosone.org/static/policies.action#sharing) **requires authors to make all data underlying the findings described in their manuscript fully available without restriction, with rare exception (please refer to the Data Availability Statement in the manuscript PDF file). The data should be provided as part of the manuscript or its supporting information, or deposited to a public repository. For example, in addition to summary statistics, the data points behind means, medians and variance measures should be available. If there are restrictions on publicly sharing data—e.g. participant privacy or use of data from a third party—those must be specified.**

**Reviewer #1: Yes**

**Reviewer #2: Yes**

**4. Is the manuscript presented in an intelligible fashion and written in standard English?

PLOS ONE does not copyedit accepted manuscripts, so the language in submitted articles must be clear, correct, and unambiguous. Any typographical or grammatical errors should be corrected at revision, so please note any specific errors here.**

**Reviewer #1: Yes**

**Reviewer #2: Yes**

**5. Review Comments to the Author

Please use the space provided to explain your answers to the questions above. You may also include additional comments for the author, including concerns about dual publication, research ethics, or publication ethics. (Please upload your review as an attachment if it exceeds 20,000 characters)**

**Reviewer #1: Well done strudy with rigorous analysis. Excellent that researchers looked at symptom onset rather than date of diagnosis for MS onset. In general therwe was little pregnancy loss. Surprislingly with 52.4% of the cohort smokers, smoking had litle impact on pregnancy loss or risl for MS. The authors explained this well. The appendix contsained a detailed review ofthe Bayesian Model- appreciated.
Although these questions will not hold publication, I wondered what was the definition of a "sensitgivity analysis", when was the study completed? and definitions for the nongynecologist would be helpful (blighteed ovum? and missed abortion?)
Well done- this may go to press**

Thank you for your review. Designing and conducting a research study necessitates making choices with regards to inclusion criteria, censoring criteria, covariates, and statistical models. In the current study, the main analysis included choices which we regarded as optimal to answer the research question. However, a range of sensitivity analyses were also conducted to further explore the impact of making slightly different choices (eg. further adjusted for smoking status). Concordance between primary results and sensitivity analyses indicated the results were robust to these changes.

Please note, 52.4% does not indicate the proportion of smokers in the population. The proportion indicates the proportion of the total population with smoking information available. Among these women, the proportion who were ever tobacco smokers was 20.1%. We have added the following to clarify this:

*Secondary analyses examined the effect of redefining the outcome date as the date of onset of symptoms (median age at onset 31.2 years, IQR 24.8-38.2), excluding women who never achieved pregnancy, (n=1,029,795; 68.0% of the cohort), or adjusting for smoking status (n=793,482; smoking data available for 52.4% of the cohort [among these women 20.1% were registered as ever tobacco smokers]), ending follow-up in the year 2000 (n=1,107,618; 73.2% of the cohort), using minimally informative priors, or using a negative binomial model with a cubic spline on the covariate age. These secondary analyses did not change the statistical significance of the results, as seen in Table 2.* **(Page 10, line 198-205)**

We have included a short description of blighted ovum and missed abortion in the manuscript: *A pregnancy loss was defined as the registered loss of a pregnancy before viability and included miscarriage, blighted ovum (ie. a pregnancy with a visible gestational sac, but no visible embryo), and missed abortion (ie. a nonviable pregnancy remaining in the uterus).* **(page 4, line 85-88)**

**Reviewer #2:**

**This is well written paper from Denmark utilizing the Danish nationwide register data. Authors studied whether pregnancy loss (miscarriage) is associated with autoimmune neurological disorders including multiple sclerosis (MS), amyotrophic lateral sclerosis, Guillain-Barré syndrome and myasthenia gravis. Study material consisted of female population aged 12 and older living in Denmark between 1977 and 2017. Findings from the main analysis and bunch of secondary and sensitivity analyses were reassuring: pregnancy loss was not significantly associated with autoimmune neurological disorder. Previous live births and higher education were negatively associated with MS while calendar year, age and family history increased the risk of MS. Conclusions are supported by the data.

Study exposure i.e. pregnancy loss was identified from the Danish Patient Register using diagnostic codes that had been previously validated. Also, an algorithm to identify same and different pregnancy episode are clinically justified. Authors write that they may not have captured early spontaneous pregnancy losses that does not require treatment at the hospitals. There is no reason to anticipate misclassification according to disease status. Also, sensitivity analysis was conducted restricting the study end into year 2000 as before that miscarriages were mainly treated at hospitals.

Study outcome was incident diagnosis recorded at the Danish Multiple Sclerosis Register with high completeness and validity. Secondary outcomes were identified from the Danish Patient Register.

Authors have conducted Bayesian Poisson regression to estimate adjusted incidence rate ratios and 95% credible intervals. Bayesian approach is justified based on rare event. However, Bayesian approach necessitates prior information on the probability of the outcome, but only sparse data existed to guide the model. A sensitivity analysis using a negative binomial model was done which did not change results substantially.

The study presents the results of the original research and have not been published elsewhere. Study material and analyses are described in sufficient detail and study reports scientifically sound results following the STROBE guidelines. Data cannot be made available due to Danish law for secondary use of register data. I have mainly minor issues where text could be improved and/or clarification added.**

Thank you for reviewing our manuscript.

**On p. 6 authors describe smoking as a proxy for unmeasured lifestyle and environmental factors possibly associated with outcome. Only women with non-missing data were included in the sensitivity analysis and sample size was limited almost to a half. Also, smoking status was only known for women after a delivery and cannot be extrapolated to women without delivery history. How this could affect the results? Would there be a possibility to identify smokers from the patient registry data e.g. ICD-10 F17? Or drug refills of varenicline ATC code N07A03? The missing information on smoking in pregnancies ending before viability seems a limitation that has not been fully addressed. Possibly associated with the outcome is an understatement as in the literature smoking has been associated both with increased risk of disease and with MS disease activity (see eg.** [**https://pubmed.ncbi.nlm.nih.gov/32259516/**](https://pubmed.ncbi.nlm.nih.gov/32259516/)**).**

Thank you for this comment. We have revised the sentence regarding “smoking is possibly associated with the outcome”: *Third, as smoking status (categories: yes/no) is associated with multiple sclerosis, we conducted an analysis including this covariate.[26]* **(Page 7, line 145-146)**

As the reviewer states, smoking status using the current definition was only known for women with a delivery after 1997 (52.4% of the population). Adjusting for smoking in this population did not change the results materially. We cannot exclude the distribution of exposure and outcomes may be different in the remaining 47.6% of women, this is stated in the limitations section. The reviewer suggests an alternative smoking definition based on ICD-10 code F17 (which would require hospitalization for smoking) or use a drug for smoking cessation (ATC: N07AB]). This method would likely underestimate smoking status, as few smokers are likely to be admitted for smoking or to take drugs against smoking. Despite underestimating the covariate, the misclassification is likely non-differential. For the reviewer we have conducted the sensitivity analysis and included it in the Supporting information as S2 Table:

| S2 Table. Association of Pregnancy loss with Multiple Sclerosis, further adjusted for smoking status defined by hospital admission for smoking (ICD-10: F17) or fulfilling prescription for a smoking cessation drug (ATC: N07AB). | | | | | |
| --- | --- | --- | --- | --- | --- |
| **Analysis**  **Pregnancy loss exposure ^a^** | **Events** | **Person-years** | **Incidence rate ^b^** | **Crude IRR ^c^ (95%CI)** | **Adjusted IRR ^c, d^ (95% CI)** |
|  | n = 1,513,544 (100%) | | | | |
| 0 | 6,654 | 36,656,369 | 1.82 | 1 | 1 |
| 1 | 807 | 2,986,620 | 2.70 | 1.49 (1.38- 1.60) | 1.03 (0.95-1.11) |
| 2 | 137 | 505,490 | 2.71 | 1.48 (1.25- 1.75) | 1.02 (0.86-1.20) |
| ≥3 non-consecutive | 12 | 61,249 | 1.96 | 1.06 (0.60- 1.73) | 0.75 (0.42-1.24) |
| Primary RPL ^e^ | 30 | 87,626 | 3.42 | 1.80 (1.24- 2.52) | 1.21 (0.83-1.71) |
| Secondary RPL ^e^ | 27 | 82,839 | 3.26 | 1.71 (1.15- 2.44) | 1.20 (0.81-1.71) |
| *Abbreviations:* ATC: Anatomical Therapeutic Chemical Classification System, ICD: International Statistical Classification of Diseases and Related Health Problems; CI, credible interval; IR, incidence rate; IRR, incidence rate ratio;  RPL: recurrent pregnancy loss  ^a^ Pregnancy loss (i.e. miscarriage) was defined hierarchically with no pregnancy losses at the lowest level and recurrent pregnancy loss at the highest level.  ^b^ Incidence rate per 10,000 person-years.  ^c^ Estimated using a Poisson model  ^d^ Estimate adjusted for the number of live births, obtained bachelor’s degree, family history of multiple sclerosis, calendar period, and age group unless otherwise stated.  ^e^ Recurrent pregnancy loss (RPL) defined as three consecutive pregnancy losses, either preceded by a delivery (secondary) or not (primary). | | | | | |

We have revised the following statement in the limitations section: *Smoking status was only known from year 1997 and onwards for women with a delivery (ie. a live- or stillbirth), and consequently the result cannot necessarily be extrapolated to women with no prior deliveries. Therefore, a post-hoc sensitivity analysis (S2 Table) explored the effect of identifying smokers by hospital diagnosis code (ICD-10: F17) or fulfilling a prescription for a smoking cessation drug (Anatomical Therapeutic Chemical Classification code: N07BA), this did not change the significance of the results.* **(Page 14, line 247-252)**

**Missing information is always a challenge in register-based studies. Complete-case analysis would restrict the sample size substantially. Modelling missing data as a separate category is an alternative, however that may result in study findings that are hard to interpret. Have you considered multiple imputation? In Table 1, unknown information on education was significantly associated with MS. Any ideas what could explain the finding? It is also uncertain where does unknown family history derive from? First degree relatives not found from the Patient Registry? Look-back period can have an impact on how reliable information can be obtained for the parents. Please explain and add into p.5 line 108 what years were available from the Danish Patient registry to identify MS in first degree relatives.**

Please note, since we the submitted the manuscript, additional educational data on the population have become available. Originally, we stated 10.3% of women had missing data in the covariate ‘educational status’. With the updated educational data, the proportion of persons with missing data in this covariate has decreased to 3.7%. This has been updated throughout the manuscript and results in slight changes in adjusted estimates (Table 1-3). After this update, unknown educational status is no longer significantly associated with MS, as seen in Table 1 in the revised manuscript.

Additionally, 9.3% had missing data in the covariate ‘family history of multiple sclerosis’. These persons had no information about one or both parents in the Civil Registration System, were kinship information has been registered since 1968 in Denmark. Missing data on family history was negatively associated with MS as seen in Table 1. We suspect persons with missing family data were often immigrants (or persons with parents not living in Denmark) and these persons may also have a lower genetical susceptibility to MS, this would explain the finding. We have added the following in the methods section: *Kinship information was available since 1968 in the Civil Registration System. As the Danish Multiple Sclerosis Register was only available for women, multiple sclerosis among first degree relatives (mother, listed father, or full sibling) was identifiable since 1977 in the Danish National Patient Register using the International Classification of Diseases and Related Health Problems, 8th revision (ICD-8), and 10^th^ revision (ICD-10) codes for multiple sclerosis: ICD-8: 340, ICD-10: G35.* **(Page 5, line 108-113)**

We did consider multiple imputation for handling missing values, however, we had concerns that missing data did not occur at random (eg. more prevalent among immigrants) and therefore would not provide additional benefit in this study. Furthermore, multiple imputation would be highly computationally intensive in Bayesian analyses. For the reviewer we have provided the risk estimates for the association between pregnancy loss and multiple sclerosis using multiple imputation in the corresponding frequentist model due to markedly lower computation time:

| **Main analysis for outcome of multiple sclerosis using Poisson model with missing values as separate categories** | |
| --- | --- |
| **Pregnancy loss** | **Adjusted IRR (95% CI)** |
| 0 | 1 |
| 1 | 1.03 (0.95-1.11) |
| 2 | 1.02 (0.86-1.20) |
| ≥3 non-consecutive | 0.81 (0.51-1.24) |
| Primary RPL | 1.18 (0.84-1.60) |
| Secondary RPL | 1.16 (0.81-1.63) |
| **Frequentist Poisson model with imputed missing values using MICE package (50 imputed datasets).** | |
| 0 | 1 |
| 1 | 0.98 (0.91-1.06) |
| 2 | 0.94 (0.79-1.12) |
| ≥3 non-consecutive | 0.69 (0.39-1.22) |
| Primary RPL | 1.16 (0.81-1.67) |
| Secondary RPL | 1.13 (0.78-1.66) |

As seen, the estimates are slightly lower using multiple imputation, however the significance of the results are unchanged. We have added a section about missing values in the limitations section: *In the cohort missing values were rare: 9.7% had missing information on one or both parents leading to unknown family history of an outcome, and 3.7% had missing information on educational status. We acknowledge this could be a potential source of bias.* **(Page 15, line 276-278)**

**-abstract says age 11 while on page 4 age 12**

Thank you. We have changed the sentence in the abstract: *This register-based cohort study, included the Danish female population age 12 or older between 1977-2017.* **(page 2, line 27-28)**

**-p3. lines 59-64 extremely long and fluctuating sentence. Please split. Also, the concept of secondary and primary pregnancy loss needs to be explained here when mentioned for the first time in the main text.**

Thank you. We have revised the sentence: *Recurrent pregnancy loss, in Denmark defined as three consecutive pregnancy losses, (although other countries use a definition of two consecutive losses), occurs for 1-2% of women trying to conceive.[14,15] Recurrent pregnancy loss can be divided into primary recurrent pregnancy loss which is not preceded by a delivery, and secondary recurrent pregnancy loss which is preceded by a delivery. Primary and secondary pregnancy loss are possibly linked to a different spectrum of diseases,[16,17] and have further been associated to specific maternal HLA alleles.[18,19]* **(page 3, line 58-64)**

**-p4 line 90 other pregnancies were identified but not directly used in analyses. What do you mean? Was used or wasn´t used?**

What is meant is that ectopic pregnancies did not count as a pregnancy loss or used as a covariate in analyses. We see this sentence is unclear and have revised it: *As complications of early pregnancy could lead to multiple hospital contacts during clinical care, an algorithm using restriction periods was used (S1 Appendix) to ascertain each pregnancy was only counted once. Therefore, additional pregnancy outcomes were also identified: ectopic pregnancy, molar pregnancy, and induced abortion, to separate individual pregnancies. The latter were identified in the Danish Register of Legally Induced Abortions.[23]* **(Page 4-5, line 91-96)**

**-p5 line 101 3 or more pregnancy losses (not fulfilling criteria for recurrent pregnancy loss) is a bit unclear. It should be possible to understand the design without S1 Table. It is crucial to understand the difference between category 3+ and recurrent primary and secondary as these are repeated in result tables and text with often slightly different working in the brackets. Couldn´t you just simply name the category ≥ 3 non-consecutive pregnancy losses?**

Thank you for this suggestion. We have changed this category to ≥3 non-consecutive pregnancy losses throughout, as suggested. *Women could increase exposure level during follow-up but not revert, and could only contribute risk time in one category at a given time during follow-up (categories: no pregnancy losses, 1 pregnancy loss, 2 pregnancy losses, ≥3 non-consecutive pregnancy losses [not fulfilling criteria for recurrent pregnancy loss], primary recurrent pregnancy loss, and secondary recurrent pregnancy loss).* **(Page 5, line 100-104)** Also changes in wording in abstract **(Page 2, line 37-38)** and results **(Page 8, line 177, line 182)**, **Table 1** and **Table 2**.

**-p5 line 105 explain the difference between date of diagnosis and onset of disease? Which one was used as date of incidence in the analysis? In p. 6 sensitivity analysis date of symptom onset was used as an event date. Is onset of disease the same as date of symptom onset? Check the wording and be consistent.**

Thank you for this comment. In the main analysis, the date of diagnosis was used as the event date. Upon diagnosis, the physician estimates the date of MS symptom onset through reviewing hospital records. This is often multiple years before the diagnosis. A sensitivity analysis explored the impact of using the date of symptom onset as the event date. We have revised the sentence: *In case the date of diagnosis or date of symptom onset was given as the calendar year, June 1 was chosen as the date of diagnosis or symptom onset.* **(Page 5, line 107-108).** Further changes to wording in abstract **(Page 2, line 36) and Table 2.**

**p.6 line 140 delete of**

This has been deleted: *The date of symptom onset was assessed and registered by the clinician upon diagnosing multiple sclerosis.* **(page 6, line 142-143)**

**p.7 line 151. Rationale for studying exposure to stillbirth is lacking. Also, the definition changed in the Danish Medical Birth Register over time from 28 weeks before 2004 to 22 weeks, how that was taken into account in the analysis?**

Both pregnancy loss and stillbirth have been associated with later cardiovascular disease (eg. <https://pubmed.ncbi.nlm.nih.gov/23536362/>). To be thorough, we also considered fetal death at higher gestational ages (stillbirth) in assessing association between pregnancy loss and MS. Analyses were adjusted for calendar year, but beyond this no additional stratification was done based on the cut-off in 2004. Findings did not show signs of any meaningful association between stillbirth and MS, and considering this, we did not see any benefit of further stratifying analyses by the cut-off year. We have revised the following sentence: *Fifth, exposure to stillbirth (categories: 0, ≥1) was examined, as stillbirth has been associated with other diseases such as cardiovascular disease.[26]* **(Page 7, line 1503-154)**

**p.14 lines 261-263 data quality for secondary outcomes in Danish Patient Register is provided. What about the validity of MS diagnosis i.e. capture of familial disease which was adjusted in the models?**

The outcome of multiple sclerosis was based the Multiple Sclerosis Register which is assumed to be virtually complete (<https://pubmed.ncbi.nlm.nih.gov/33128351/>). The Multiple Sclerosis Register is partially based on the National Patient Register; however, records are assessed by a specialist before being entered into the register (and others sources of data may also be used). The Multiple Sclerosis Register was only available for women, and therefore family history of MS diagnosis was based on a MS diagnosis in the National Patient Register. The validity of these diagnosis codes have been validated against the Multiple Sclerosis Register, showing a completeness of 92.8% and validity of 95.1% (<https://onlinelibrary.wiley.com/doi/10.1111/j.1600-0404.2011.01558.x>). Linking to first degree relatives was done using the Civil Registration System. The validity of correct linkage has to our knowledge not been tested, likely due to ethical concerns of knowing which listed fathers are not the true biological fathers. The maternal linking is likely very accurate as it occurs right after birth. We added the following in the limitations section: *Family linkage was based on the Civil Registration System and has to our knowledge not been validated. Correct assignment of the mothers is likely very accurate as is occurs right after birth. Family history of multiple sclerosis was based on diagnosis codes in the Danish National Patient Register which have been validated against the Danish Multiple Sclerosis Registry and shown a completeness of 92.8% and validity of 95.1%.[36]* **(Page 15, line 271-276)**

**S1 Figure. Should the red distribution be 0.25-4.00 in the figure? Mean being IRR=1 upper credible limit cannot be 0.40, right?**

Thank you for noticing this mistake. The upper CI should be ~4 and not 0.4. This has now been corrected. **(S1 Fig.)**

6. PLOS authors have the option to publish the peer review history of their article ([what does this mean?](https://journals.plos.org/plosone/s/editorial-and-peer-review-process#loc-peer-review-history)). If published, this will include your full peer review and any attached files.

Yes, please publish peer-review history

If you choose “no”, your identity will remain anonymous but your review may still be made public.


**Do you want your identity to be public for this peer review?** For information about this choice, including consent withdrawal, please see our [Privacy Policy](https://www.plos.org/privacy-policy).

Reviewer #1: **Yes:** Heidi Maloni PhD

Reviewer #2: **Yes:** Maarit Leinonen

[NOTE: If reviewer comments were submitted as an attachment file, they will be attached to this email and accessible via the submission site. Please log into your account, locate the manuscript record, and check for the action link "View Attachments". If this link does not appear, there are no attachment files.]

While revising your submission, please upload your figure files to the Preflight Analysis and Conversion Engine (PACE) digital diagnostic tool, <https://pacev2.apexcovantage.com/>. PACE helps ensure that figures meet PLOS requirements. To use PACE, you must first register as a user. Registration is free. Then, login and navigate to the UPLOAD tab, where you will find detailed instructions on how to use the tool. If you encounter any issues or have any questions when using PACE, please email PLOS at [figures@plos.org](mailto:figures@plos.org). Please note that Supporting Information files do not need this step.

**OTHER CHANGES TO MANUSCRIPT**

Additional data on education for the population was made available during the review of this manuscript. This data has now been included, reducing the proportion with missing data in this covariate from 10.3% to 3.7%. Therefore, all analyses have been run again and adjusted estimates in Table 1-3 have been updated. The changes are minor, mostly on the second decimal point of the estimate, with no changes to the significance of the results. **(Table 1-3)**
